# Synthetic multiantigen MVA vaccine COH04S1 and variant-specific derivatives protect Syrian hamsters from SARS-CoV-2 Omicron subvariants

**DOI:** 10.1038/s41541-023-00640-y

**Published:** 2023-03-16

**Authors:** Felix Wussow, Mindy Kha, Taehyun Kim, Minh Ly, Marcal Yll-Pico, Swagata Kar, Mark G. Lewis, Flavia Chiuppesi, Don J. Diamond

**Affiliations:** 1 Department of Hematology and Transplant Center, City of Hope National Medical Center, Duarte, CA 91010 USA; 2 Bioqual, Rockville, MD USA

**Keywords:** Live attenuated vaccines, Viral infection, Live attenuated vaccines, Antibodies

## Abstract

Emerging SARS-CoV-2 Omicron subvariants continue to disrupt COVID-19 vaccine efficacy through multiple immune mechanisms including neutralizing antibody evasion. We developed COH04S1, a synthetic modified vaccinia Ankara vector that co-expresses Wuhan-Hu-1-based spike and nucleocapsid antigens. COH04S1 demonstrated efficacy against ancestral virus and Beta and Delta variants in animal models and was safe and immunogenic in a Phase 1 clinical trial. Here, we report efficacy of COH04S1 and analogous Omicron BA.1- and Beta-specific vaccines to protect Syrian hamsters from Omicron subvariants. Despite eliciting strain-specific antibody responses, all three vaccines protect hamsters from weight loss, lower respiratory tract infection, and lung pathology following challenge with Omicron BA.1 or BA.2.12.1. While the BA.1-specifc vaccine affords consistently improved efficacy compared to COH04S1 to protect against homologous challenge with BA.1, all three vaccines confer similar protection against heterologous challenge with BA.2.12.1. These results demonstrate efficacy of COH04S1 and variant-specific derivatives to confer cross-protective immunity against SARS-CoV-2 Omicron subvariants.

## Introduction

Despite unprecedented mass vaccination and availability of effective COVID-19 vaccines, SARS-CoV-2 remains a threat to human health due to the continuous emergence of variants of concern (VOC) with ability to evade vaccine-induced immunity. Following emergence of Alpha (B.1.1.7), Beta (B.1.351), Gamma (P.1), and Delta (B.1.617)^1,2^, Omicron subvariants have emerged as predominant SARS-CoV-2 VOC, which includes BA.1, BA.2, and BA.2 sub-lineages BA.2.12.1, BA.4/BA.5, and BA.2.75^3–8^, as well as the currently dominating BA.2-derived Omicron subvariant XBB.1.5^9^. Omicron subvariants have exceptional capacity to evade neutralizing antibodies (NAb) due to numerous mutations in the spike (S) protein that dramatically exceed S mutations of other earlier occurring VOC^3–9^, thereby posing a unique challenge for COVID-19 vaccination. Several studies report reduced clinical effectiveness against Omicron variants by approved COVID-19 vaccines^10–13^, which were designed to elicit protective immunity based on the S protein of the Wuhan-Hu-1 reference strain^14–18^. Waning COVID-19 vaccine efficacy against major VOC can be counteracted by repeated booster vaccination^10–12^, while updated COVID-19 booster vaccines with altered or variant-matched antigen design have been developed to specifically enhance the stimulation of cross-protective immunity against emerging SARS-CoV-2 VOC^19,20^.

We developed COVID-19 vaccine COH04S1, a multiantigen synthetic modified vaccinia Ankara (sMVA) vector that co-expresses Wuhan-Hu-1-based S and nucleocapsid (N) antigens^21,22^. COH04S1 afforded protection against SARS-CoV-2 ancestral virus and Beta and Delta variants in Syrian hamster and non-human primate models and was safe and immunogenic in a Phase 1 clinical trial in healthy adults^22–24^. COH04S1 is currently being tested in multiple Phase 2 clinical trials in healthy volunteers and in cancer patients (NCT4639466, NCT4977024). In this report, we assessed the efficacy of COH04S1 and analogous Omicron BA.1 or Beta-specific vaccines to protect against Omicron subvariants in Syrian hamsters. We found that the three vaccines elicited strain-specific antibody responses, while all three vaccines conferred efficacy to protect hamsters from weight loss, lower respiratory tract infection, and lung pathology following challenge with Omicron BA.1 or BA.2.12.1. In contrast to the Beta-specific vaccine, the BA.1-specific vaccine conferred consistently improved efficacy compared to COH04S1 to protect against homologous challenge with Omicron BA.1. However, neither the Beta- nor the BA.1-specific vaccine afforded significantly improved efficacy compared to COH04S1 to protect against heterologous challenge with BA.2.12.1. These results demonstrate that COH04S1 and analogous variant-specific derivatives confer cross-protective immunity against SARS-CoV-2 Omicron subvariants.

## Results

### Vaccine-elicited ancestral- and variant-specific humoral immune responses

**Fig. 1:**
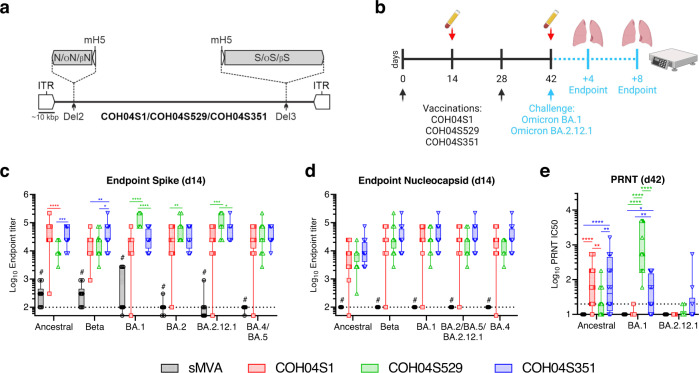
COH04S1 and Omicron BA.1 and Beta sequence-modified vaccines elicit strain-specific antibody responses against SARS-CoV-2 ancestral virus and VOC. **a**. Vaccine constructs. COH04S1, COH04S529, and COH04S351 are sMVA-vectored COVID-19 vaccines co-expressing S and N antigens based on the Wuhan-Hu-1 reference strain or Omicron BA.1 (Ο) or Beta (β) variants, respectively. The antigen sequences were inserted into the MVA deletion sites 2 (Del2) and 3 (Del3) as indicated. **b**. Study design. Hamsters were vaccinated twice with COH04S1 (*n* = 20), COH04S351 (*n* = 20), or COH04S529 (*n* = 20) by intramuscular route as indicated (black arrows). Hamsters vaccinated with empty sMVA vector (*n* = 10), or unvaccinated hamsters (*n* = 10) were used as controls. Blood samples were collected at day 14 and 42 (red arrows) after the first and second vaccination. COH04S1-, COH04S529-, and COH04S351-vaccinated hamsters were challenged intranasally at day 42 with Omicron subvariants BA.1 or BA.2.12.1 (*n* = 10/group). sMVA control animals were challenged with BA.1. Unvaccinated controls were challenged with BA.2.12.1. Post-challenge body weight changes were recorded daily for 8 days. Lung tissue and nasal turbinates for viral load measurements and lung histopathology were collected at days 4 and 8 post-challenge (*n* = 5/group/timepoint). IgG endpoint titers. S-specific (**c**) and N-specific (**d**) binding antibody titers to ancestral virus, Beta and Omicron subvariants BA.1, BA.2, BA.2.12.1, BA.4, and BA.5 were measured in serum samples of vaccine and control groups at day 14 (d14) post-prime vaccination via ELISA. Dotted lines indicate lower limit of detection (LLOD). # indicates significantly higher IgG titers in vaccine groups compared to controls. **e**. NAb titers. NAb titers were measured in serum samples of vaccine and control groups at day 42 (d42) post-second vaccination via PRNT assay against ancestral SARS-CoV-2 (WA/01), and Omicron BA.1 and BA.2.12.1. Dotted line indicates LLOD. Values below the LLOD are indicated as half the LLOD. Data are presented as box plots extending from 25th to 75th percentiles, with lines indicating medians, and whiskers going from minimum to maximum values. Two-way ANOVA with Tukey’s multiple comparison test was used in c-e following log transformation. *0.05 < *p* < 0.01, **0.01 < *p* < 0.001, ***0.001 < *p* < 0.0001, ****p < 0.0001. When not indicated, differences are not significant (*p* > 0.05).

Syrian hamsters were vaccinated with COH04S1 or analogous vaccine constructs with Omicron BA.1 or Beta (B.1.351) sequence-modified S and N antigens, termed COH04S529 or COH04S351, respectively (Fig. 1a, b and Supplementary Table 1). Hamsters were vaccinated twice in a four-week interval by intramuscular route. All three sMVA vaccines elicited potent post-prime binding antibody responses to S and N antigens of SARS-CoV-2 ancestral virus, Beta variant, and Omicron variants BA.1, BA.2, BA.2.12.1 and BA.4/5 (Fig. 1c, d). COH04S1 and the Beta-specific COH04S351 stimulated significantly higher ancestral-specific S antibody titers than the BA.1-specific COH04S529 vaccine. Consistent with the modified antigen sequences, the Beta-specific COH04S351 vaccine elicited significantly higher Beta-specific S antibody responses than COH04S1 and COH04S529, and the BA.1-specific COH04S529 vaccine elicited significantly higher BA.1-specific S antibody responses than COH04S1 and COH04S351. In addition, COH04S529 vaccination resulted in increased Omicron BA.2-specific S antibody titers compared to COH04S1 vaccination as well as increased BA.2.12.1-specific S antibody titers compared to COH04S1 or COH04S351 vaccination. In contrast, BA.4/BA.5-specific S antibody titers were comparable between the three vaccine groups (Fig. 1c). No significant differences were observed for ancestral- and variant-specific N antibody titers between the three vaccine groups (Fig. 1d).

At 2 weeks post-vaccination prior to virus challenge, NAb responses were measured by plaque reduction neutralization titer (PRNT) assay against ancestral SARS-CoV-2 (USA-WA1/2020) and Omicron BA.1 or BA.2.12.1. NAb responses against ancestral SARS-CoV-2 were measured in all three vaccine groups, although ancestral-specific NAb titers in COH04S1- and COH04S351-vaccinated hamsters were significantly higher than those in COH04S529-vaccinated animals (Fig. 1e). Consistent with the Omicron immune evasion capacity, BA.1-specific NAb responses in COH04S1-vaccinated animals were either very low or undetectable. In contrast, COH04S529-vaccinated hamsters showed potent Omicron BA.1-specific NAb titers that significantly exceeded BA.1-specific NAb titers measured in COH04S1- and COH04S351-vaccinated animals (Fig. 1e), consistent with the BA.1-specific vaccine modification. BA.1-specific NAb titers measured in COH04S351-vaccinated animals were of intermediate level compared to those measured in COH04S1- and COH04S529-vaccinated hamsters. In contrast to BA.1-specific NAb responses, BA.2.12.1-specific NAb titers measured in all three vaccine groups were either very low or undetectable, indicating limited cross-neutralizing activity against Omicron BA.2.12.1 by the vaccine-elicited responses. Interestingly, BA.2.12.1-specific NAb titers measured in COH04S351-vaccinated animals were slightly elevated compared to those measured in COH04S1- and COH04S529-vaccinated hamsters, although these differences in BA.2.12.1-specific responses between the three vaccine groups were not statistically significant. These results demonstrate that COH04S1 and Omicron BA.1- and Beta-specific vaccine derivatives elicit strain-specific antibody responses against ancestral SARS-CoV-2, Beta variant, and Omicron subvariants.

### Vaccine protection against Omicron BA.1 or BA.2.12.1 virus-induced weight loss

**Fig. 2:**
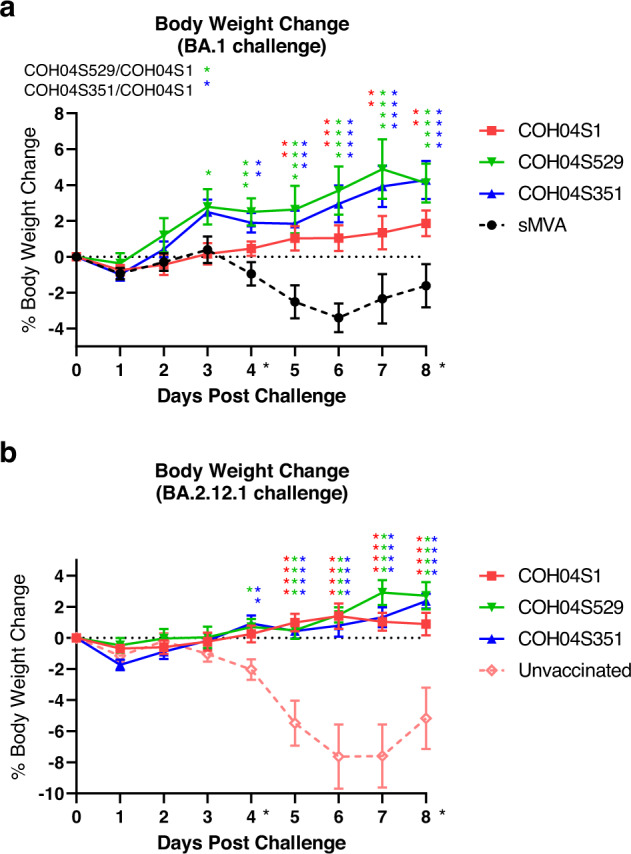
COH04S1 and Omicron BA.1- and Beta-modified vaccines protect hamsters from weight loss following challenge with Omicron BA.1 or BA.2.12.1. Body weight of COH04S1, COH04S529-, COH04S351-vaccinated animals was measured daily for 8 days following challenge with Omicron variants BA.1 (**a**) and BA.2.12.1 (**b**). Hamsters vaccinated with empty sMVA vector or unvaccinated hamsters were used as controls. Weight loss compared to day 0 is reported as mean ± SEM. Two-way ANOVA followed by Tukey’s multiple comparison test was used to compare group mean values at each timepoint following log transformation. Color-coded asterisks indicate significant difference compared to controls unless specified. *0.05 < *p* < 0.01, **0.01 < *p* < 0.001, ***0.001 < *p* < 0.0001, ****p < 0.0001. When not indicated, differences are not significant (*p* > 0.05). Black asterisks indicate time of sacrifice (*n* = 5/group).

At two-week post vaccination, hamsters were intranasally challenged with Omicron variant BA.1 or BA.2.12.1 and body weight was measured for 8 days. Hamsters vaccinated with sMVA without inserted antigens and unvaccinated hamsters were used as controls. Control animals showed progressive weight loss following challenge with BA.1 or BA.2.12.1, with maximum weight loss between 4-8% at day 6 post vaccination. In contrast, body weight of hamsters vaccinated with COH04S1 or the variant-specific vaccines remained relatively stable or increased gradually over the 8-day observation period following challenge with either BA.1 or BA.2.12.1 (Fig. 2a, b). Notably, both COH04S529- and COH04S351-vaccinated animals showed consistently higher body weight than COH04S1-vaccinated hamsters following challenge with BA.1, suggesting improved vaccine efficacy through both the BA.1 and Beta-specific antigen modification to protect against BA.1-induced weight loss, although these differences in weight between vaccine groups were only significant at day 3 post-challenge (Fig. 2a). In contrast, similar body weight changes were measured for all three vaccine groups following challenge with BA.2.12.1 (Fig. 2b), indicating similar levels of protection against BA.2.12.1-indcued weight loss by all three vaccines. These results show that COH04S1 and Omicron BA.1 and Beta sequence-modified vaccines protect hamsters from weight loss following challenge with Omicron BA.1 and BA.2.12.1.

### Vaccine protection against Omicron BA.1 or BA.2.12.1 respiratory tract infection

**Fig. 3:**
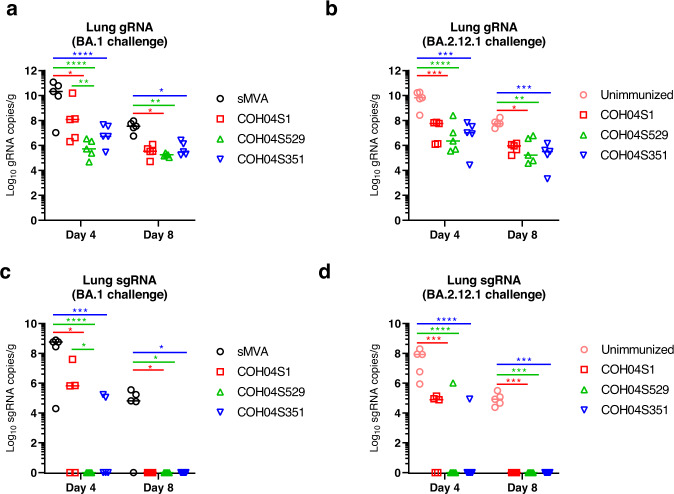
COH04S1 and Omicron BA.1- and Beta-modified vaccines protect hamsters from lower respiratory tract infection following virus challenge with Omicron BA.1 or BA.2.12.1. SARS-CoV-2 genomic RNA (gRNA, **a**, **b**) and sub-genomic RNA (sgRNA, **c**, **d**) copies were quantified by qPCR in lung tissue of COH04S1, COH04S351, and COH04S529 vaccine and control groups at days 4 and 8 following challenge with Omicron subvariants BA.1 (**a**, **c**) and BA.2.12.1 (**b**, **d**). Lines indicate median RNA copies. Two-way ANOVA followed by Tukey’s multiple comparison test was used following log transformation. *0.05 < *p* < 0.01, **0.01 < *p* < 0.001, ***0.001 < *p* < 0.0001, ****p < 0.0001. When not indicated, differences are not significant (*p* > 0.05).

**Fig. 4:**
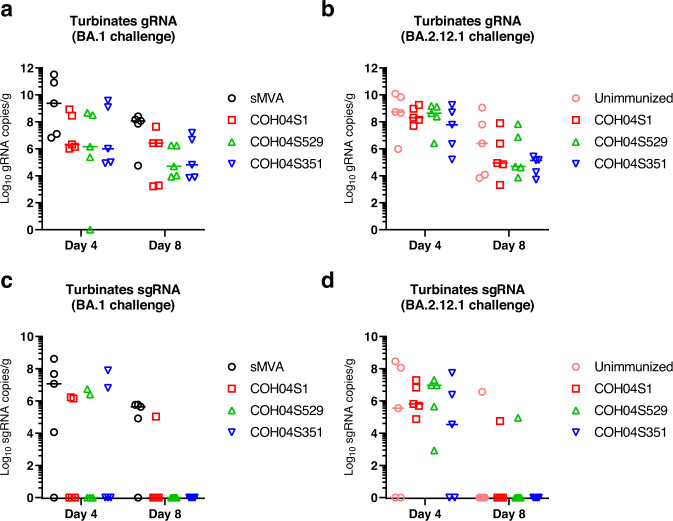
Viral loads at upper respiratory tracts in COH04S1-, COH04S529-, and COH04S351-vaccinated hamsters following challenge with Omicron BA.1 or BA.2.12.1. SARS-CoV-2 genomic RNA (gRNA, **a**, **b**) and sub-genomic RNA (sgRNA, **c**, **d**) copies were quantified by qPCR in nasal turbinates of COH04S1, COH04S529, and COH04S351 vaccine and control groups at days 4 and 8 following challenge with Omicron subvariants BA.1 (**a**, **c**) and BA.2.12.1 (**b**, **d**). Lines indicate median RNA copies. One-way ANOVA followed by Tukey’s multiple comparison test was used following log transformation. When not indicated, differences are not significant (*p* > 0.05).

At days 4 and 8 post challenge, viral loads were measured in lung tissue and nasal turbinates by quantification of SARS-CoV-2 genomic RNA (gRNA) and sub-genomic RNA (sgRNA) to assess the magnitude of total and replicating virus at lower and upper respiratory tracts. Compared to the high lung viral loads measured in control animals, significantly reduced gRNA and sgRNA levels were measured in the lungs of all three vaccine groups at day 4 and 8 following virus challenge with BA.1 or BA.2.12.1 (Fig. 3a–d), demonstrating potent efficacy of all three vaccines to control lower respiratory tract infection by BA.1 or BA.2.12.1. In addition, in contrast to all or most control animals, all animals of the vaccine groups had undetectable sgRNA in the lungs at day 8 following BA.1 or BA.2.12.1 virus challenge (Fig. 3c, d), indicating complete control of lower respiratory tract infection. Notably, unlike COH04S351-vaccinated animals, COH04S529-vaccinated animals had significantly reduced lung viral loads compared to COH04S1-vaccinated animals at day 4 following challenge with BA.1 (Fig. 3a, c), consistent with improved immediate viral control of BA.1 lower respiratory tract infection through the BA.1-specific vaccine adaptation. Moreover, in contrast to COH04S1- and COH04S351-vaccinated animals, all COH04S529-vaccinated animals had undetectable sgRNA at day 4 following BA.1 challenge (Fig. 3c). No significant differences in lung viral loads were observed between the three vaccine groups at day 8 following challenge with BA.1 (Fig. 3a, c). In addition, no significant differences in lung viral loads were observed between the three vaccine groups at day 4 or 8 following BA.2.12.1 virus challenge (Fig. 3b, d) indicating comparable vaccine efficacy to protect against lower respiratory infection caused by BA.2.12.1. Compared to the potent vaccine efficacy to protect against Omicron BA.1 or BA.2.12.1 infection in the lower respiratory tract, the vaccine efficacy to control infection of the Omicron variants in the upper respiratory tract was less evident (Fig. 4a–d). While viral loads measured in nasal turbinates of all three vaccine groups following virus challenge with BA.1 were consistently lower than those of controls (Fig. 4a, c), these differences in nasal viral loads were not statistically significant. Moreover, no evident differences in nasal viral loads were observed between the vaccine groups and controls following challenge with BA.2.12.1 (Fig. 4b, d). The precise reason for the low vaccine efficacy to prevent upper respiratory tract infection is unclear, although this may suggest limited protective mucosal immunity at the respiratory epithelium. Other explanations such as an excessive viral challenge dose, as suggested by the relatively high viral loads in both the vaccine and control groups, may also account for this observation. These results demonstrate efficacy of COH04S1 and Omicron BA.1- and Beta sequence-modified vaccines to protect hamsters against lower respiratory tract infection by Omicron BA.1 and BA.2.12.1.

### Vaccine protection against Omicron BA.1 or BA.2.12.1 virus-induced lung pathology

**Fig. 5:**
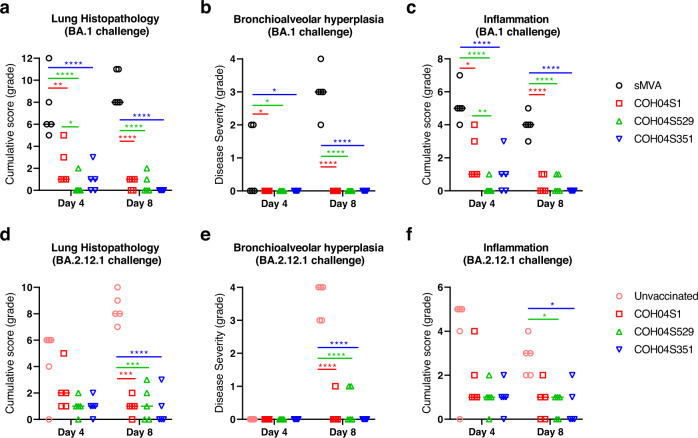
COH04S1 and Omicron BA.1- and Beta-modified vaccines protect hamsters from lung pathology following challenge with Omicron BA.1 or BA.2.12.1. Hematoxylin/eosin-stained lung sections of COH04S1-, COH04S529, and COH04S351-vaccinated hamsters and control animals at days 4 and 8 following challenge with SARS-CoV-2 BA.1 (**a**–**c**) or BA.2.12.1 (**d**–**f**) variants were evaluated by a board-certified pathologist and microscopic findings were graded based on severity on a scale from 1 to 5 (Supplementary Table 3). Cumulative pathology score of all histopathologic findings (**a**, **d**), grading of bronchioalveolar hyperplasia disease severity (**b**, **e**), and severity of lung inflammatory microscopic findings (**c**, **f**) are shown. Lines indicate median values. Two-way ANOVA followed by Tukey’s multiple comparison test was used following log transformation. *=0.05 < *p* < 0.01, **=0.01 < *p* < 0.001, ***=0.001 < *p* < 0.0001, ****=*p* < 0.0001. When not indicated, differences are not significant (*p* > 0.05).

Lung pathology in all three vaccine groups following virus challenge with BA.1 or BA.2.12.1 was significantly reduced when compared to controls (Fig. 5a–f, Supplementary Fig. 1), indicating efficacy of all three vaccines to protect hamsters from BA.1- and BA.2.12.1-induced lung injury. In addition, a subset of control hamsters had moderate bronchioalveolar hyperplasia (i.e., type II pneumocyte hyperplasia) at day 4 following BA.1 virus challenge, and all control animals had moderate to high-grade bronchioalveolar hyperplasia at day 8 following viral challenge with BA.1 or BA.2.12.1 (Fig. 5b, e). In contrast, bronchioalveolar hyperplasia was undetectable in animals of all three vaccine groups at day 4 and 8 following viral challenge with BA.1 or BA.2.12.1, with only one or two exceptions in the COH04S1 and COH04S529 vaccine groups that showed low-grade bronchioalveolar hyperplasia (Fig. 5b, e). Lung pathology in all three vaccine groups appeared mostly associated with inflammation, which was detectable at low levels in all vaccine groups at day 4 and 8 following virus challenge with BA.1 or BA.2.12.1, albeit at significantly reduced levels across all vaccine groups compared to controls (Fig. 5c, f), confirming efficacy of all three vaccines to protect against BA.1 or BA.2.12.1-induced lung pathology. Notably, in contrast to COH04S351-vaccinated animals, COH04S529-vaccinated animals had significantly reduced lung pathology and inflammation compared to COH04S1-vaccinated animals at day 4 following challenge with BA.1 (Fig. 5a, c), indicating improved protection by the BA.1-specific vaccine to control BA.1-induced lung pathology during early phase after viral challenge. No significant differences in lung pathology were observed between the three vaccine groups at day 8 following BA.1 virus challenge (Fig. 5a–c). Furthermore, no significant differences in lung pathology, hyperplasia, or inflammation were observed between the vaccine groups at day 4 or 8 following virus challenge with BA.2.12.1 (Fig. 5d–f), indicating comparable efficacy of all three vaccines to protect against BA.2.12.1-induced lung pathology. These results demonstrate that COH04S1 and Omicron BA.1 and Beta sequence-modified vaccine derivatives protect hamsters from lung pathology following virus challenge with Omicron subvariants BA.1 and BA.2.12.1.

## Discussion

This report demonstrates that sMVA-based COVID-19 vaccine COH04S1 co-expressing Wuhan-Hu-1 S and N antigens and analogous Omicron BA.1 (COH04S529) and Beta (COH04S351) sequence-modified vaccines protect Syrian hamsters from SARS-CoV-2 Omicron subvariants BA.1 and BA.2.12.1. These results complement previous observations in Syrian hamster and non-human primate models demonstrating potent efficacy of COH04S1 and COH04S351 to protect against SARS-CoV-2 ancestral virus and Beta and Delta VOC^22,23^. These animal studies provide evidence that COH04S1 and variant-specific derivatives have potent capacity to confer cross-protective immunity against emerging SARS-CoV-2 VOC, including Omicron subvariants.

While repeated booster vaccination can counteract waning efficacy observed with first-generation Wuhan-Hu-1-based COVID-19 vaccines^10–12^, variant-specific vaccines have been developed to enhance vaccine efficacy against Omicron subvariants. Two Omicron BA.4/BA.5-specific mRNA booster vaccines have been recently authorized by the FDA^20,25,26^, while the EMA has authorized mRNA boosters with BA.1- and BA.4/BA.5-specific modifications. Studies in hamsters and non-human primates provide evidence that Wuhan-Hu-1-based COVID-19 vaccines can protect against Omicron B.1.1.529 and its BA.1 subvariant^27–30^. Yet, the impact of Omicron-specific sequence alterations to enhance COVID-19 vaccine efficacy is debatable^25,26,30–32^. Moreover, the advantage of the bivalent mRNA booster vaccine containing BA.4/BA.5- and Wuhan-Hu-1-based S antigens over a booster by the original Wuhan-Hu-1-based mRNA vaccine to stimulate cross-neutralizing responses against Omicron subvariants remains controversial^20,33^. Our observations in Syrian hamsters demonstrating efficacy of COH04S1 to protect against Omicron BA.1 and BA.2.12.1 supports that Wuhan-Hu-1-based COVID-19 vaccines have the capacity to confer potent cross-protective immunity against Omicron variants. Unlike the Beta-specific COH04S351 vaccine, the BA.1-specific COH04S529 conferred consistently improved efficacy compared to COH04S1 to protect against weight loss, lower respiratory tract infection, and lung pathology during early phase (at day 4) following homologous challenge with BA.1, while all three vaccines conferred comparable levels of protection against heterologous challenge with BA.2.12.1. These results suggest that Omicron-specific antigen modification can improve the efficacy against homologous Omicron strains, while it does not appear to be an effective strategy to enhance protection against heterologous Omicron strains. Updated Omicron-specific booster vaccines may not provide a significant advantage over first-generation Wuhan-Hu-1-based COVID-19 vaccines to confer long-term protection against evolving Omicron subvariants. However, heterologous-prime boost immunization strategies were not investigated, which could potentially be a more effective approach to further enhance cross-protective immunity conferred by the vaccines.

Whether the observed efficacy of COH04S1 or the variant-adapted vaccines to protect against Omicron BA.1 and BA.2.12.1 was mediated solely by S-specific responses or by a combination of S and N-specific responses remains unclear. The results that COH04S529 elicited more potent BA.1-specific antibody responses and provided greater protection against BA.1 virus challenge than COH04S1 is consistent with an important protective role of humoral immunity. Interestingly, despite the stimulation of low-to-undetectable BA.1-specific NAb responses by COH04S1 and low-to-undetectable BA.2.12.1-specific NAb responses by all three vaccines, all three vaccines provided potent protection against BA.1 and BA.2.12.1 virus challenge. While these findings may indicate that only low NAb titers are required to protect against Omicron variants in hamsters, these findings may also indicate that other responses besides NAb contribute to the efficacy of COH04S1 and its sequence-modified vaccine derivatives. This may include humoral responses promoting Fc-mediated effector functions as well as T cell responses to both S and N antigens. A recent study with Wuhan-Hu-1-based mRNA vaccines in hamsters shows that an antigen combination composed of S and N confers superior efficacy compared to S alone to protect against Omicron BA.1^34^, supporting the use of a dual antigen design to confer protective immunity against Omicron variants. Other studies support potential benefits of N as a vaccine antigen^35–37^. Whether the efficacy observed with COH04S1 or the variant-specific vaccines against BA.1 and BA.2.12.1 extends to other Omicron subvariants remains to be addressed. The antigenic similarity of the S and N proteins between Omicron variants evolved from BA.2 suggests that the vaccines may have the capacity to similarly protect against more recently emerged Omicron variants.

The N antigen was included in COH04S1 primarily based on the rationale to broaden the induction of T cells, which are known to be less susceptible to antigen variation than NAb and therefore considered a critical second line of defense to provide long-term protective immunity against SARS-CoV-2^38–44^. Our previous studies in mice and non-human primates and our Phase 1 clinical trial in healthy adults demonstrates potent capacity of COH04S1 to stimulate S and N-specific T cells^21,22,24,38^. Importantly, T cell responses to both the S and N antigens elicited in COH04S1-vaccinated healthy adults maintain potent cross-reactivity to Delta and Omicron BA.1 variants for up to six months post-vaccination^38^, whereas COH04S1-elicited NAb response, as shown for other COVID-19 vaccines^45^, decrease and confer reduced neutralizing activity against Delta and Omicron BA.1. While we did not assess T cell responses in the current study due to the limitations to evaluate cellular responses in the hamster model, these prior studies in animals and healthy adults suggest that T cells responses may have contributed to the efficacy of COH04S1 and its allied forms to protect against Omicron BA.1 and BA.2.12.1 in hamsters. T cell responses may become critically important for vaccine protection against SARS-CoV-2 when NAb responses are suboptimal, as suggested by our observations in this study. Furthermore, because of the higher conservation of the N protein compared to the S protein, N-specific T cells may have greater potential than S-specific T cells to confer cross-reactive immunity against SARS-CoV-2 VOC.

Additional studies are needed to identify immune correlates of protection that are associated with COH04S1 vaccine protection and to specifically address the contribution of vaccine-elicited S and N-specific responses to confer cross-protective immunity. Nonetheless, our current data in sum combined with our previous results in animal models demonstrates potent efficacy of COH04S1 and analogous variant-adapted vaccines to protect against multiple SARS-CoV-2 VOC, including Omicron subvariants. Importantly, COH04S1 is the most advanced clinically evaluated MVA-vectored COVID-19 vaccine and the only COVID-19 vaccine combining S and N that is actively investigated in Phase 2 clinical trials. COH04S1 may therefore be a valuable alternative to the currently available single-antigen FDA-approved COVID-19 vaccines based on adenoviral vectors or mRNA to improve cross-protective immunity against emerging SARS-CoV-2 VOC.

## Methods

### Vaccine vectors

COH04S1 is a double-plaque purified virus isolate derived from the previously described sMVA-N/S vector (NCBI Accession# MW036243) with N and S antigen sequences inserted into the MVA deletion sites 2 (Del2) and 3 (Del3), respectively^21,22,46^. COH04S1 co-expresses full-length, unmodified S and N antigen sequences based on the Wuhan-Hu-1 reference strain (NCBI Accession# NC_045512). COH04S351 is a double-plaque purified virus isolate with analogous vaccine construction compared to the original COH04S1 vector and co-expresses modified S and N antigen sequences based on the B.1.351 Beta variant (Supplementary Table 1)^2,47^. COH04S529 is a non-plaque purified virus isolate with analogous vaccine construction compared to the original COH04S1 sMVA-N/S vaccine vector and co-expresses modified S and N antigen sequences based on the Omicron BA.1 variant (Supplementary Table 1). COH04S1, COH04S351, and COH04S529 were generated using the sMVA platform consisting of three bacterial artificial chromosome-cloned synthetic DNA fragments (sMVA fragments F1-F3) covering the MVA genome sequence published by Antoine and colleagues^21,46^. The antigen sequences were inserted into the sMVA fragments F1 and F3 by *En passant* mutagenesis in GS1783 *E. coli* cells^21,48^. The modified sMVA fragments F1 and F3 with inserted antigen sequences in Del2 or Del3, respectively, were co-transfected together with the unmodified sMVA fragment F2 into baby hamster kidney cells to initiate the reconstitution of recombinant sMVA virus in the presence of fowl pox virus as a helper virus^21^. Virus stocks of the vaccine vectors and sMVA control vector were produced using chicken embryo fibroblasts (CEF) and prepared by 36% sucrose cushion ultracentrifugation and virus resuspension in 1 mM Tris-HCl (pH 9). Virus stocks were stored at −80 °C and titrated on CEF by immunostaining of viral plaques at 16–24 h post infection using polyclonal vaccinia antibody (9503-2057, Bio-Rad, dilution 1:2000)^21^. Viral stocks were validated for antigen insertion and expression by PCR, sequencing, and Immunoblot.

### Hamster study

In life portion of the hamster studies were carried out at Bioqual Inc. (Rockville, MD). Eighty 6–8 weeks old Syrian hamsters were randomly assigned to the groups, with 5 females and 5 males in each challenge group. Hamsters were intramuscularly vaccinated four weeks apart with 1 × 10^8^ plaque forming units (PFU) of COH04S1, COH04S529, COH04S351 or sMVA virus stocks diluted in phosphate-buffered saline (PBS) or left unvaccinated. Blood samples were collected 2 weeks after the first vaccine dose, and two weeks after the second dose. At this latter time point animals were challenged intranasally (50 µl/nare) with 4.8 × 10^4^ Median Tissue Culture Infectious Dose [TCID50] of SARS-CoV-2 BA.1 (BEI resources NR-56486 LOT#: 70049695, titered by BEI resources using Calu-3 cells) or with 5.16 × 10^4^ TCID50 of SARS-CoV-2 BA.2.12.1 (BEI resources NR-56782 LOT#: 70052277, titered using Calu-3 cells). Body weight was recorded daily for 8 days. Hamsters were humanely euthanized for lung and turbinate samples collection at day 4 (*n* = 5/group) and day 8 (*n* = 5/group) post-challenge. All animal studies were conducted in compliance with local, state, and federal regulations and were approved by Bioqual (protocol 20-163) and City of Hope (protocol 20087) Institutional Animal Care and Use Committees.

### Binding antibody detection

SARS-CoV-2-specific binding antibodies in hamster serum samples were detected by indirect ELISA using purified ancestral-specific, Beta-specific, Omicron BA.1-, BA.2-, BA.2.12.1-, and BA.4-specific S proteins (Sino Biological 40589-V08B1, 40588-V07E9, 40589-V08H26, 40589-V08H28, 40589-V08H34, 40589-V08H32), and purified ancestral-specific, Beta-specific, Omicron BA.1-, BA.2-, and BA.4-specific N proteins (Sino Biological 40588-V08B, 40589-V08B7, 40588-V07E34, 40588-V07E35, 40588-V07E37). S and N mutations included in the antigens used for ELISA are indicated in Supplementary Table 2. 96-well plates were coated with 100 μl/well of protein at a concentration of 1 µg/ml in PBS and incubated overnight at 4^o^C. Plates were washed 5× with wash buffer (0.1% Tween-20/PBS), then blocked with 250 µl/well of blocking buffer (0.5% casein/154 mM NaCl/10 mM Tris-HCl [pH 7.6]) for 2 h at room temperature. After washing, threefold diluted heat-inactivated serum in blocking buffer was added to the plates and incubated 2 h at room temperature. After washing, anti-Hamster IgG(H + L) HRP secondary antibody (Southern Biotech 6061-05) was diluted 1:1000 in blocking buffer and added to the plates. After 1 h incubation, plates were washed and developed with 1 Step TMB-Ultra (Thermo Fisher 34029). The reaction was stopped with 1 M H_2_SO_4_ and plates were read on FilterMax F3 (Molecular Devices). Endpoint titers were calculated as the highest dilution to have an absorbance >0.100 nm.

### Neutralization assay

NAb were measured by PRNT using ancestral SARS-CoV-2 (USA-WA1/2020 strain), or BA.1, or BA.2.12.1 variants. Ancestral SARS-CoV-2 stock was generated using Vero-E6 cells infected with seed stock virus obtained from Kenneth Plante at UTMB (lot # TVP 23156). BA.1 stock (BIOQUAL Lot # 122121-700) was originally received from Emory (B.1.1.529 PP3P1 hCoV19/EHC_C19_2811C 12/9/2021) and expanded in Calu-3 cells. BA.2.12.1 stock was obtained from BEI resources (NR-56782) and produced on Calu-3 cells. Vero E6 cells (ATCC, CRL-1586) were seeded in 24-well plates at 175,000 cells/well in DMEM/10% FBS/Gentamicin. Serial threefold serum dilutions were incubated in 96-well plates with 30 PFU of SARS-CoV-2 for 1 h at 37 °C. The serum/virus mixture was transferred to Vero-E6 cells and incubated for 1 h at 37 °C. After that, 1 ml of 0.5% methylcellulose media was added to each well and plates were incubated at 37 °C for three days. Plates were washed, and cells were fixed with methanol. Crystal violet staining was performed, and plaques were recorded. IC50 titers were calculated as the serum dilution that gave a 50% reduction in viral plaques in comparison to control wells.

### Genomic RNA quantification

SARS-CoV-2 gRNA copies per gram of tissue were quantified by qRT-PCR assay using primer/probe sequences binding to a conserved region of SARS-CoV-2 N gene. Viral RNA was extracted with RNA-STAT 60 (Tel-test B)/chloroform, precipitated and resuspended in RNAse-free water. SensiFAST Probe Lo‑ROX One‑Step Kit (Bioline BIO-78005) was used following manufacturer instructions. All samples were tested in triplicate. The control RNA was prepared to contain 10^6^ to 10^7^ copies per 3 µl. Eight 10-fold serial dilutions of control RNA were prepared using RNAse-free water. Duplicate samples of each dilution were prepared. Amplification was carried out using an Applied Biosystems 7500 Sequence detector. Amplification conditions were 48 °C for 30 min, 95 °C for 10 min followed by 40 cycles of 95 °C for 15 seconds, and 1 min at 55 °C. Copies of RNA/ml were calculated by extrapolation from the standard curve. Primer/probe sequences: 5′-GAC CCC AAA ATC AGC GAA AT-3′; 5′-TCT GGT TAC TGC CAG TTG AAT CTG-3′; and 5′-FAM-ACC CCG CAT TAC GTT TGG TGG ACC-BHQ1-3′.

### Subgenomic RNA quantification

SARS-CoV-2 sgRNA copies were assessed through quantification of N gene mRNA by qRT-PCR^22,23^. Briefly, SARS-CoV-2 RNA was extracted from tissues using TRIzol, precipitated and resuspended in RNAse-free water. For quantification, the standard curve of a plasmid containing a cDNA copy of the N gene mRNA target was used. Applied Biosystems 7500 Real-Time PCR System was used for amplification with the following program: 48 °C for 30 min, 95 °C for 10 min followed by 40 cycles of 95 °C for 15 s, and 1 min at 55 °C. The number of copies of RNA per ml was calculated by extrapolation from the standard curve and multiplying by the reciprocal of 0.2 ml extraction volume. Primer/probe sequences: 5′-CGA TCT CTT GTA GAT CTG TTC TC-3′; 5′-GGT GAA CCA AGA CGC AGT AT-3′; 5′-FAM- TAA CCA GAA TGG AGA ACG CAG TGG G-BHQ-3′.

### Histopathology

Histopathological evaluation of hamster lung sections was performed by Experimental Pathology Laboratories, Inc. (Sterling, VA). At necropsy organs were collected and placed in 10% neutral buffered formalin for histopathologic analysis. Tissues were processed through to paraffin blocks, sectioned once at approximately 5 microns thickness, and stained with hematoxylin/eosin. Board certified pathologists were blinded to the vaccine groups and controls were used as a comparator. Histopathological findings were assigned a severity score between 1 (minimal) and 5 (severe) (Supplementary Table 3).

### Statistical analysis

Statistical analyses were performed using Prism 8 (GraphPad, v8.3.0). One-way ANOVA and two-way ANOVA with Tukey’s multiple comparison test were used for statistical evaluation after logarithmic transformation. The significance level for each test is indicated in the figure legends.

### Reporting summary

Further information on research design is available in the Nature Research Reporting Summary linked to this article.

## Supplementary information

Supplemental Information

REPORTING SUMMARY

## Data Availability

The datasets generated during and/or analyzed during the current study are available from the corresponding authors on reasonable request.
