# Supplementary material for: Synthetic multiantigen MVA vaccine COH04S1 and variant-specific derivatives protect Syrian hamsters from SARS-CoV-2 Omicron subvariants
Source: NPJ Vaccines. 2023 Mar 16;8:41. doi: 10.1038/s41541-023-00640-y (PMC10018591; doi:10.1038/s41541-023-00640-y)
Supplement: Supplementary file 1 — Supplemental Information [file 41541_2023_640_MOESM1_ESM.pdf]

Synthetic multiantigen MVA vaccines COH04S1 and variant-specific derivatives protect Syrian hamsters from SARS-CoV-2 Omicron subvariants

Supplementary Information

Supplementary Table 1. Related to Figure 1. S and N antigen-specific mutations in COH04S529 and COH04S351 vaccine sequences.

		Substitutions/Deletions (Δ) to Wuhan-Hu-1 reference strain	
Vaccine	Strain	Spike (S)	Nucleocapsid (N)
COH04S529	B.1.529	A67V, Δ69-70 (HV), T95I, G142D, Δ143-145 (VYY), Δ211 (N), L212I, INS214 (EPE), G339D, S371L, S373P, S375F, K417N, N440K, G446S, S477N, T478K, E484A, Q493R, G496S, Q498R, N501Y, Y505H, T547K, D614G, H655Y, N679K, P681H, N764K, D796Y, N856K, Q954H, N969K, L981F	P13L, Δ31-33 (ERS), R203K, G204R
COH04S351	B.1.351	L18F, D80A, D215G, Δ242-244, R246I, N501Y, E484K, K417N, D614G, A701V	T205I

Supplementary Table 2. Related to Figure 1. Mutations in S and N antigens for ELISA testing

Strain	*VOC*	*Spike*[1, 2]	*Nucleocapsid*[1]	*Notes*
B.1.351	Beta	L18F, D80A, D215G, Δ242-244 (LAL), R246L, K417N, E484K, N501Y, D614G, A701V	T205I	
B.1.1.529 BA.1	Omicron BA.1	A67V, Δ69-70 (HV), T95I, G142D, Δ143-145 (VYY), Δ211 (N), L212I, INS214 (EPE), G339D, S371L, S373P, S375F, K417N, N440K, G446S, S477N, T478K, E484A, Q493R, G496S, Q498R, N501Y, Y505H, T547K, D614G, H655Y, N679K, P681H, N764K, D796Y, (F817P), N856K, (A892P), (A899P), (A942P), Q954H, N969K, L981F, (K986P), (V987P)	P13L, Δ31-33 (ERS), R203K, G204R	
B.1.1.529 BA.2	Omicron BA.2	T19I, L24S, Δ25-27 (PPA), G142D, V213G, G339D, S371F, S373P, S375F, T376A, D405N, R408S, K417N, N440K, S477N, T478K, E484A, Q493R, Q498R, N501Y, Y505H, D614G, H655Y, N679K, P681H, N764K, D796Y, (F817P), (A892P), (A899P), (A942P), Q954H, N969K, (K986P), (V987P)	P13L, Δ31-33 (ERS), R203K, G204R, S413R	BA.2, BA.5, BA.2.12.1, BA.2.75 share the same N mutations
B.1.1.529 BA.2.12.1	Omicron BA.2.12.1	T19I, L24S, del25-27 (PPA), G142D, V213G, G339D, S371F, S373P, S375F, T376A, D405N, R408S, K417N, N440K, L452Q, S477N, T478K, E484A, Q493R, Q498R, N501Y, Y505H, D614G, H655Y, N679K, P681H, S704L, N764K, D796Y, (F817P), (A892P), (A899P), (A942P), (Q954H), (N969K), (K986P), (V987P)	P13L, Δ31-33 (ERS), R203K, G204R, S413R	BA.2, BA.5, BA.2.12.1, BA.2.75 share the same N mutations
B.1.1.529 BA.4	Omicron BA.4	T19I, L24S, Δ25-27 (PPA), Δ69-70 (HV), G142D, V213G, G339D, S371F, S373P, S375F, T376A, D405N, R408S, K417N, N440K, L452R, S477N, T478K, E484A, F486V, Q498R, N501Y, Y505H, D614G, H655Y, N679K, P681H, N764K, D796Y, (F817P), (A892P), (A899P), (A942P), Q954H, N969K, (K986P), (V987P)	P13L, Δ31-33 (ERS), P151S, R203K, G204R, S413R	BA.4 and BA.5 share the same S mutations

[1]Substitutions/Deletions (Δ)/Insertions (INS) to Wuhan-Hu-1 reference strain

[2]in parenthesis mutations added to stabilize the trimer in pre-fusion conformation

Supplementary Table 3. Related to Figure 5. Scoring parameters used to evaluate hamster lung histopathology

Grade	Severity	Findings
1	Minimal	histopathologic change ranging from inconspicuous to barely noticeable but so minor, small, or infrequent as to warrant no more than the least assignable grade. For multifocal or diffusely-distributed lesions, this grade was used for processes where less than approximately 10% of the tissue in an average high-power field was involved. For focal or diffuse hyperplastic/hypoplastic/ atrophic lesions, this grade was used when the affected structure or tissue had undergone a less than approximately 10% increase or decrease in volume.
2	Mild	histopathologic change that is a noticeable but not a prominent feature of the tissue. For multifocal or diffusely-distributed lesions, this grade was used for processes where between approximately 10% and 25% of the tissue in an average high-power field was involved. For focal or diffuse hyperplastic/hypoplastic/atrophic lesions, this grade was used when the affected structure or tissue had undergone between an approximately 10% to 25% increase or decrease in volume.
3	Moderate	histopathologic change that is a prominent but not a dominant feature of the tissue. For multifocal or diffusely-distributed lesions, this grade was used for processes where between approximately 25% and 50% of the tissue in an average high-power field was involved. For focal or diffuse hyperplastic/hypoplastic/atrophic lesions, this grade was used when the affected structure or tissue had undergone between an approximately 25% to 50% increase or decrease in volume.
4	Marked	histopathologic change that is a dominant but not an overwhelming feature of the tissue. For multifocal or diffusely-distributed lesions, this grade was used for processes where between approximately 50% and 95% of the tissue in an average high-power field was involved. For focal or diffuse hyperplastic/hypoplastic/atrophic lesions, this grade was used when the affected structure or tissue had undergone between an approximately 50% to 95% increase or decrease in volume.
5	Severe	histopathologic change that is an overwhelming feature of the tissue. For multifocal or diffusely-distributed lesions, this grade was used for processes where greater than approximately 95% of the tissue in an average high-power field was involved. For focal or diffuse hyperplastic/hypoplastic/atrophic lesions, this grade was used when the affected structure or tissue had undergone a greater than approximately 95% increase of decrease in volume.

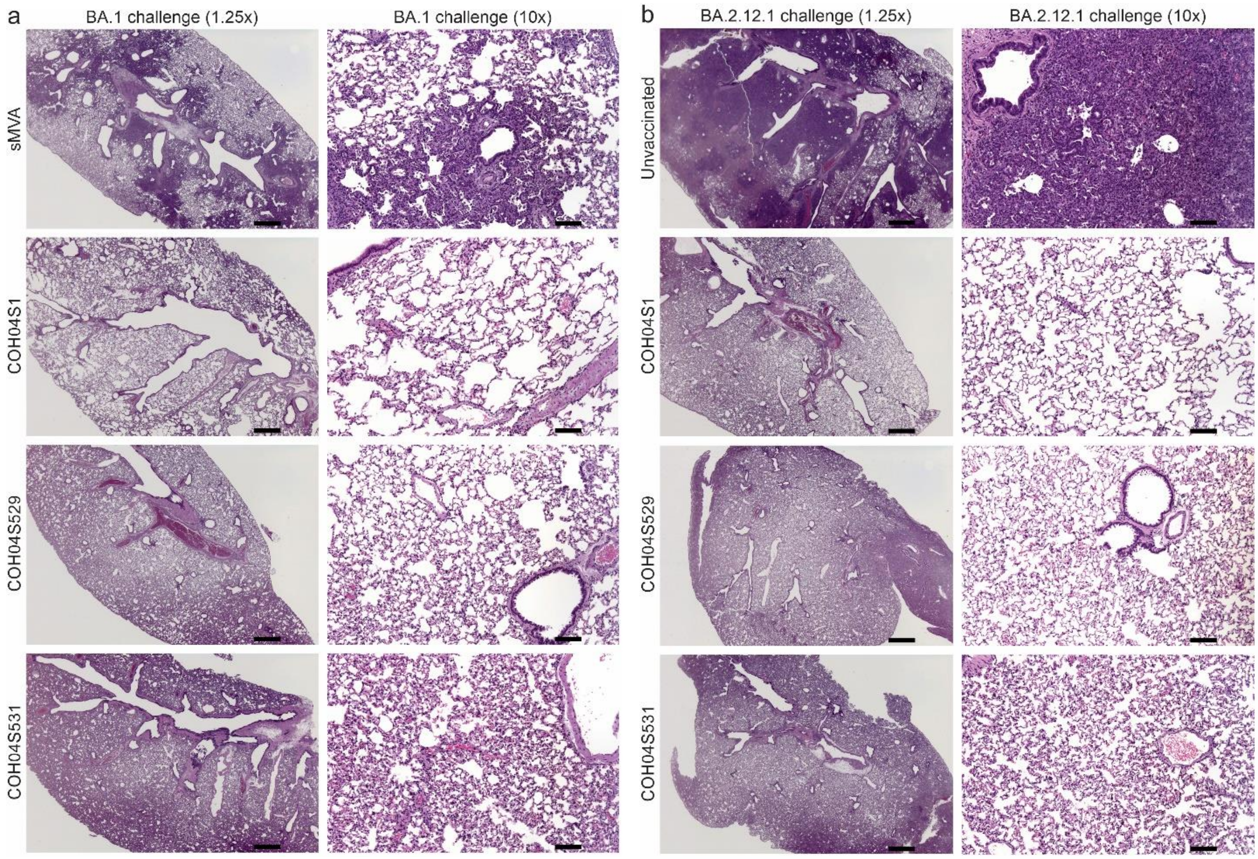

Supplementary Figure 1. Related to Figure 5. Histopathological findings in lungs of COH04S1-, COH04S529- and COH04S351-vaccinated hamsters following virus challenge with Omicron BA.1 and BA.2.12.1. Shown are representative images (1.25x and 10x magnification) of histopathological findings in hematoxylin/eosin-stained lung sections of COH04S1-, COH04S529, and COH04S351-vaccinated hamsters and sMVA or unvaccinated control animals at day 8 post-challenge with SARS-CoV-2 BA.1 (a) or BA.2.12.1 (b) variants. Control animals show extensive areas with deeply basophilic and concentrated findings affecting the majority of the lung. Darker areas in COH04S351 and COH04S529 animals in (a) are considered insufficiently inflated lungs and not SARS-CoV-2-related. Open airways were observed in COH04S1-, COH04S529, and COH04S351-vaccinated hamsters. Scale bar=800 µm for 1.25x images. Scale bar=100 µm for 10x images.
