# Supplementary material for: Synthetic multiantigen MVA vaccine COH04S1 and variant-specific derivatives protect Syrian hamsters from SARS-CoV-2 Omicron subvariants
Source: NPJ Vaccines. 2023 Mar 16;8:41. doi: 10.1038/s41541-023-00640-y (PMC10018591; doi:10.1038/s41541-023-00640-y)
Supplement: Supplementary file 2 — REPORTING SUMMARY [file 41541_2023_640_MOESM2_ESM.pdf]

nature portfolio

Corresponding author(s): Felix Wussow

Last updated by author(s): Feb 23, 2023

Reporting Summary

Nature Portfolio wishes to improve the reproducibility of the work that we publish. This form provides structure for consistency and transparency in reporting. For further information on Nature Portfolio policies, see our Editorial Policies and the Editorial Policy Checklist.

Statistics

For all statistical analyses, confirm that the following items are present in the figure legend, table legend, main text, or Methods section.

n/a	Confirmed	
☐	☒	The exact sample size (*n*) for each experimental group/condition, given as a discrete number and unit of measurement
☐	☒	A statement on whether measurements were taken from distinct samples or whether the same sample was measured repeatedly
☐	☒	The statistical test(s) used AND whether they are one- or two-sided<br>*Only common tests should be described solely by name; describe more complex techniques in the Methods section.*
☒	☐	A description of all covariates tested
☐	☒	A description of any assumptions or corrections, such as tests of normality and adjustment for multiple comparisons
☐	☒	A full description of the statistical parameters including central tendency (e.g. means) or other basic estimates (e.g. regression coefficient) AND variation (e.g. standard deviation) or associated estimates of uncertainty (e.g. confidence intervals)
☐	☒	For null hypothesis testing, the test statistic (e.g. *F*, *t*, *r*) with confidence intervals, effect sizes, degrees of freedom and *P* value noted<br>*Give P values as exact values whenever suitable.*
☒	☐	For Bayesian analysis, information on the choice of priors and Markov chain Monte Carlo settings
☒	☐	For hierarchical and complex designs, identification of the appropriate level for tests and full reporting of outcomes
☒	☐	Estimates of effect sizes (e.g. Cohen's *d*, Pearson's *r*), indicating how they were calculated

Our web collection on statistics for biologists contains articles on many of the points above.

Software and code

Policy information about availability of computer code

Data collection	RT-qPCR data was collected with Applied Biosystems 7500<br>Absorbance was collected with SoftMax Pro Software
Data analysis	RT-qPCR data was analyzed with Applied Biosystems 7500<br>GraphPad Prism 8.3.0 and Microsoft Excel were used for data and statistical analyses

For manuscripts utilizing custom algorithms or software that are central to the research but not yet described in published literature, software must be made available to editors and reviewers. We strongly encourage code deposition in a community repository (e.g. GitHub). See the Nature Portfolio guidelines for submitting code & software for further information.

Data

Policy information about availability of data

All manuscripts must include a data availability statement. This statement should provide the following information, where applicable:

- Accession codes, unique identifiers, or web links for publicly available datasets
- A description of any restrictions on data availability
- For clinical datasets or third party data, please ensure that the statement adheres to our policy

The datasets generated during and/or analyzed during the current study are available from the corresponding authors on reasonable request.

Human research participants

Policy information about studies involving human research participants and Sex and Gender in Research.

Reporting on sex and gender	n/a
Population characteristics	n/a
Recruitment	n/a
Ethics oversight	n/a

Note that full information on the approval of the study protocol must also be provided in the manuscript.

Field-specific reporting

Please select the one below that is the best fit for your research. If you are not sure, read the appropriate sections before making your selection.

☒ Life sciences ☐ Behavioural & social sciences ☐ Ecological, evolutionary & environmental sciences

For a reference copy of the document with all sections, see nature.com/documents/nr-reporting-summary-flat.pdf

Life sciences study design

All studies must disclose on these points even when the disclosure is negative.

Sample size	Each group consisted of n=10 F/M hamsters. For viral load analysis n=5 hamsters/group were euthanized at days 4 and 8 post-challenge. This study design was based on our previous study in hamsters (Wussow et al., iScience 2022. DOI: 10.1016/j.isci.2022.104457).
Data exclusions	BA.2.12.1 PRNT analysis shown in fig 1e includes n=10/group. The additional n=10 animals/group were analyzed but not included in the figure because, due to limited amount of serum, the starting dilution analyzed was higher than in all other samples. Results were comparable when 10 or 20 animals/group were used.
Replication	Viral loads were tested in triplicates. ELISA and PRNT were tested in duplicates. Technical replicates presented minimal differences and were averaged.
Randomization	Animals were assigned to the study randomly and balanced by sex.
Blinding	Animals were assigned a number and assays were performed with blinding when possible. In some assays, blinding to investigator and operators was not possible given the involvement in both sample preparation and analysis. Histopathology analysis was performed by a blinded board-certified Pathologist.

Reporting for specific materials, systems and methods

We require information from authors about some types of materials, experimental systems and methods used in many studies. Here, indicate whether each material, system or method listed is relevant to your study. If you are not sure if a list item applies to your research, read the appropriate section before selecting a response.

Materials & experimental systems

n/a	Involved in the study	
☐	☒	Antibodies
☐	☒	Eukaryotic cell lines
☒	☐	Palaeontology and archaeology
☐	☒	Animals and other organisms
☒	☐	Clinical data
☒	☐	Dual use research of concern

Methods

n/a	Involved in the study	
☒	☐	ChIP-seq
☒	☐	Flow cytometry
☒	☐	MRI-based neuroimaging

Antibodies

Antibodies used	anti-Hamster IgG(H+L) HRP secondary antibody (Southern Biotech 6061-05).<br>Rabbit anti Vaccinia Virus polyclonal IgG (BioRad 9503-2057)

Validation	Southern Biotech 6061-05 was validated by the vendor by ELISA and used in published studies (Wussow et al., iScience 2022. DOI: 10.1016/j.isci.2022.104457; Chiuppesi et al., npj vaccines 2022. DOI:10.1038/s41541-022-00436-6).<br>BioRad 9503-2057 was used in published studies (Manuel, E.R. et al. Virology 2010. DOI: 10.1016/j.virol.2010.04.015; Chiuppesi et al., 2020 Nat. Comm. DOI: 10.1038/s41467-020-19819-1).

Eukaryotic cell lines

Policy information about cell lines and Sex and Gender in Research

Cell line source(s)	Vero E6 cells (ATCC, CRL-1586); Calu-3 (ATCC, HTB-55); CEF (chicken embryo fibroblasts, Charles River); BHK-21 (ATCC, CCL-10).
Authentication	None of the cell lines were validated in-house.
Mycoplasma contamination	Cell lines were not tested for Mycoplasma contamination in house.
Commonly misidentified lines (See ICLAC register)	none

Animals and other research organisms

Policy information about studies involving animals; ARRIVE guidelines recommended for reporting animal research, and Sex and Gender in Research

Laboratory animals	Female and male Syrian golden hamsters (Envigo), 6-8 weeks old.
Wild animals	none
Reporting on sex	Sex was balanced across groups. Sex-based analysis was not performed given that in our previously published report we observed only minor differences between sexes in vaccine immunogenicity and protective efficacy when hamsters were challenged with ancestral SARS.CoV.2 or Beta and Delta variants.
Field-collected samples	none
Ethics oversight	The study was approved by: Bioqual (protocol 20-163) and City of Hope (protocol 20087) Institutional Animal Care and Use Committees (IACUC).

Note that full information on the approval of the study protocol must also be provided in the manuscript.
